# Heme Oxygenase-1 Contributes to Dampening Proinflammatory Activation in the Human Microglial Cell Line HMC3 and Controls the Transcription Factor IRF5

**DOI:** 10.3390/biom16071028

**Published:** 2026-07-14

**Authors:** Anna Lisa Furfaro, Paola Mancini, Mario Passalacqua, Jasmin Ortolan, Caterina Ivaldo, Sara Tirendi, Stefania Vernazza, Cristina d’Abramo, Luca Giliberto, Mariapaola Nitti

**Affiliations:** 1 Department of Experimental Medicine, University of Genoa, I-16132 Genova, Italy; annalisa.furfaro@unige.it (A.L.F.); paola.mancini@edu.unige.it (P.M.); mario.passalacqua@unige.it (M.P.); sara.tirendi@edu.unige.it (S.T.); stefania.vernazza@unige.it (S.V.); 2 Department of Pharmacy, University of Genoa, I-16132 Genova, Italy; jasmin.ortolan@edu.unige.it; 3 Department of Surgical and Diagnostic Sciences, University of Genoa, I-16132 Genova, Italy; caterina.ivaldo@unige.it; 4 IRCCS Azienda Ospedaliera Metropolitana (IRCCS-AOM), I-16132 Genova, Italy; 5 Litwin-Zucker Center for the Study of Alzheimer’s Disease, Institute of Molecular Medicine—The Feinstein Institutes for Medical Research, Northwell, New Hyde Park, NY 11040, USA; cdabramo@northwell.edu (C.d.); lgiliberto@northwell.edu (L.G.); 6 Zucker School of Medicine at Hofstra/Northwell, Hempstead, NY 11549, USA; 7 Department of Neurology, Zucker School of Medicine at Hofstra/Northwell, Hempstead, NY 11549, USA

**Keywords:** neuroinflammation, neurodegeneration, oxidative stress, HO-1, NRF2, interferon regulatory factor 5, microglia, TLR4, LPS

## Abstract

Neuroinflammation is recognized as a pivotal factor in promoting neurodegeneration. However, the molecular mechanisms underlying the dysregulation of proinflammatory signaling in microglial cells remain largely elusive. A prominent role has been ascribed to the TLR4 receptor and its downstream targets, among which the transcription factor IRF5 has recently gained attention. Furthermore, the enzyme HO-1 is known to play a crucial role in mediating an anti-inflammatory phenotype in microglia. This study aims to investigate whether IRF5 is a target of the anti-inflammatory activity of HO-1. Using the human microglial cell line HMC3 and validating the results in RAW264.7 macrophage-like cells, via RT-qPCR, Western blotting, and immunofluorescence, we demonstrated that HO-1 contributes to the resolution of LPS-induced proinflammatory activation. Notably, cell exposure to a pharmacological inhibitor of HO-1 increases IRF5 mRNA expression, while treatment with Hemin, able to increase HO-1 expression, significantly reduces IRF5 phosphorylation. This work suggests a possible crosstalk between HO-1 and IRF5, proposing new druggable molecular targets to counteract neuroinflammation.

## 1. Introduction

The enzyme heme oxygenase 1 (HO-1) is a crucial player among the molecular mechanisms of cell defense against stressors [1]. HO-1 gene transcription, indeed, is induced in response to oxidative stress, radiation exposure or infections and belongs to the mechanisms of cell survival and adaptation [2,3]. It carries out the degradation of heme groups and generates carbon monoxide (CO); biliverdin, further metabolized by biliverdin reductase (BVR) into bilirubin; and iron, whose toxicity is generally prevented by the parallel induction of ferritin [4,5].

The anti-inflammatory role of HO-1 has been largely studied in vasculature, where the induction of HO-1 promotes the anti-inflammatory phenotype in endothelial cells, counteracting cardiovascular diseases [6,7,8,9]. Furthermore, the activity of HO-1 has been extensively investigated in macrophages, proving that its induction reduces proinflammatory profiles and promotes a resolving phenotype [2,10]. This has been demonstrated in degenerative conditions associated with inflammation, where the lack of HO-1 is detrimental such as in acute radiation injury [11], ischemic-reperfusion injury [12] or intestinal inflammation [13], as well as in the tumor microenvironment, where the overexpression of HO-1 shapes a pro-tumor microenvironment [14,15]. In this context, most of the protective and defensive activities of HO-1 are attributed to the generation of CO [16,17] and bilirubin [18,19].

Even though the expression of HO-1 has a protective biological meaning in cells, its use as biomarker of diseases has also been proposed. Indeed, the induction of HO-1 also represents a stress condition in cells and can be used in monitoring the progression of diseases, as hypothesized in malignant cancers or in atherosclerosis [20,21,22].

In the nervous system the effect of HO-1 induction in terms of protection or damage of neuronal cells has been debated [23,24], but it is now largely recognized that the expression of HO-1 in innate immune cells and in particular in microglia cells is linked to a reduction in neuroinflammation and the prevention of neuronal loss [25,26,27].

The activation of microglial cells in the central nervous system (CNS) has beneficial roles, enabling the resolution of tissue damage in response to a variety of stressors. Nonetheless, quenching the proinflammatory activation and acquiring a resolving phenotype is pivotal to completing the defensive program against inflammation [28,29]. In fact, the dysregulation of regulatory mechanisms and the chronic activation of microglia promote tissue damage and are pivotal in the onset and progression of different neurodegenerative pathologies [29,30,31]. However, the molecular mechanisms underlying the loss of control of inflammatory response are largely unknown.

Among the proinflammatory signals, the stimulation of Toll-like receptor 4 (TLR4) plays a key role in response to infections, hypoxia, toxins and proteotoxic stress. The dysregulation of TLR4-dependent pathways has been proven to be involved in different neurodegenerative pathologies [32,33,34,35], and the role played by the downstream transcription factor interferon regulatory factor 5 (IRF5) has been highlighted [36,37]. IRF5 is known as one of the prominent genetic risk factors for autoimmune diseases [38,39] and inflammatory bowel diseases [40,41]. Notably, growing evidence shows the role of IRF5 in favoring a hyperinflammatory phenotype in macrophages, increasing, for instance, vulnerability of atherosclerotic plaques [20,42], and it has been proposed as a molecular target to counteract inflammatory diseases [43]. Furthermore, IRF5 has been identified as a crucial mechanism leading to microglial dysregulation in a mouse model of multiple sclerosis [44] or in tissue damage in ischemic stroke [45].

In this work we investigated the role of HO-1 in the regulation of TLR4-dependent proinflammatory activation in microglia cells and the possible crosstalk with IRF5.

Our work provides new insights into the molecular mechanisms that could be modulated to counteract neuronal loss associated with neuroinflammation.

## 2. Materials and Methods

### 2.1. Cell Culture and Treatments

The human HMC3 microglia cell line from ATTC (CRL-3304) was cultured in EMEM (Sigma-Aldrich, Milan, Italy) supplemented with 10% Fetal Bovine Serum (FBS) (Euroclone, Milan, Italy), 1% penicillin/streptomycin (Sigma-Aldrich) and 1% MEM (Sigma-Aldrich), subcultured twice a week, and maintained in a humidified incubator at 37 °C with 5% CO_2_. Before treatments, cells were starved for 2 h in EMEM without FBS and MEM and then treated for 1–24 h with 100 ng/mL LPS and cotreated with 10 µM tin mesoporphyrin IX (SnMP) or 5 µM Hemin, at specific time points. All the treatments did not show toxicity, as verified by the MTT test (Supplementary Figure S1a).

The murine RAW264.7 macrophage-like cell line from ATCC (TIB-71) was cultured in Low-Glucose DMEM (Euroclone) supplemented with 10% FBS (Euroclone), 1% penicillin/streptomycin (Sigma-Aldrich), 4 mM glutamine (Sigma-Aldrich), maintained in 5% CO_2_ humid atmosphere and subcultured every 2 days at 1:5. Before treatments, cells were starved for 2 h in Low-Glucose DMEM without FBS and then treated for 1–24 h with 100 ng/mL LPS and cotreated with 10 µM SnMP and 10 µM Hemin at specific time points. The treatments did not show toxicity, as verified by the MTT test (Supplementary Figure S1b). All reagents for the treatments were purchased from Sigma-Aldrich.

### 2.2. Small Interfering RNA

Small interfering RNA silencing was performed by using specifics pool of oligonucleotides against human HO-1 (On-Target Plus SMART pool human HO-1; Dharmacon, Horizon Discovery, UK) as already described [14]. Briefly, HMC3 cells were transfected with 100 nmol of siRNA (siHO-1) for 24 h by using Lipofectamine 2000 (Invitrogen—Life Technologies, Carlsbad, CA, USA). Cells were exposed to 100 ng/mL LPS for 3, 6 or 24 h. The level of mRNA expression of HO-1 was monitored to verify the efficiency of silencing. Specificity was proved by using a non-targeting silencing pool of oligonucleotides (ON-TARGETplus Non-targeting Control siRNAs pool, Dharmacon). mRNA extraction was performed as described later.

### 2.3. Immunoblotting

At the end of treatments, cells were washed with PBS, and total protein extraction was performed by using RIPA buffer [50 mM Trizma hydrochloride, pH 7.4 (SigmaAldrich), 150 mM NaCl (Carlo Erba Reagents, Milan, Italy), 1% Igepal CA-630 (Sigma-Aldrich), 0.1% sodium dodecyl sulphate (Sigma-Aldrich), supplemented with 1 mM phenyl-methane-sulfonyl fluoride (Sigma-Aldrich), 1% Phosphatase Inhibitor Cocktail 3 (Sigma-Aldrich) and with 1X Protease Inhibitors (Roche Diagnostics, Milan, Italy)] following standard protocol, as previously described [46].

Protein concentration was determined by a bicinchoninic acid assay (BCA, Pierce, ThermoScientific, Rockford, IL, USA), following the manufacturer’s instructions.

SDS-PAGE was performed following standard protocol, as previously described [47]. Blotting membranes were incubated with primary antibody (rabbit anti-HO-1, 1:1000, Abcam ab189491, Cambridge, UK) overnight at 4 °C.

After the incubation of specific secondary antibody conjugated with horseradish peroxidases (anti-rabbit 1:5000, Cell Signaling, Danvers, MA, USA), the bands were detected by means of an enhanced chemiluminescence system (ECL-PLUS system, ThermoScientific). The expression of target proteins was determined relative to the expression of tubulin used as a housekeeping protein (mouse anti-tubulin, Abcam, 1:10,000; secondary antibody anti-mouse 1:10,000, Invitrogen, ThermoScientific). The developed films were analyzed using specific software (GelDoc; Quntity One v. 4.2.0. BIO-RAD, Milan, Italy).

### 2.4. Immunofluorescence

Cells were seeded and treated in 8-well CultureSlides (Falcon, Corning, Tewksbury, MA, USA). At the end of treatments, cells were fixed and permeabilized with ice-cold methanol for 15 min. After blocking with 5% normal goat serum (NGS)/0.1% Triton/PBS, cells were incubated with rabbit polyclonal anti-HO-1 (1:100, Abcam, ab189491) or rabbit polyclonal anti p-IRF5 (Ser437, 1:500, Invitrogen, ThermoScientific, PA5-106093) in a humidified chamber overnight at 4 °C. After 3 washes with PBS, the cells were incubated with secondary anti-rabbit 568 (1:500, ThermoScientific) in PBS at room temperature for 1 h. The nuclei were stained with DAPI (1:1000) for 10 min at room temperature.

The slide was mounted using Mowiol (Polysciences, Inc., Hirschberg an der Bergstrasse, Germany), and the images were acquired using a Nikon AX R Laser-Scanning Confocal Microscope (Nikon Europe, The Netherlands). The quantification of HO-1 and p-IRF5 signals was performed by using Fiji software by measuring the corrected total cell fluorescence (CTCF). At least 4 fields per sample were quantified in each experiment, and at least 3 experiments were performed. To confirm HO-1 and p-IRF5 signal specificity, some samples were incubated with normal rabbit IgG (1:1000, Millipore, Sigma-Aldrich, 12–370) instead of specific antibodies and revealed minimal signals (see Supplementary Figures S2 and S3).

### 2.5. RT-qPCR

Total RNA extraction was performed by using the TRIzol reagent (Invitrogen) following the manufacturer’s instructions.

RNA concentration and purity were measured by using a NanoDrop DN-100 spectrophotometer machine (NanoDrop Thermo Fischer Technologies, ThermoScientific). Specifically, 2 μL/sample was utilized. The RNA purity was evaluated by the ratio of absorbance 260/280. A ratio between 1.8 and 2.0 is considered pure, without contamination from free nucleotides, ssDNA and dsDNA.

RNA (600 ng) was reverse-transcribed by using SuperScriptTM II Reverse Transcriptase (Invitrogen) and random hexamer primers following the manufacturer’s instruction, and the obtained cDNA was amplified via polymerase chain reaction (PCR).

For quantitative PCR (qPCR), diluted cDNA was amplified with a PCR Applied Biosystems QuantStudio (Applied Biosystem, Thermo Scientific, Milan, Italy) by using 1X Syber Green Master Mix (Luna Universal qPCR Master Mix, Euroclone) and 0.25µM of specific primers provided by Sigma-Aldrich and listed in Table 1. Samples were pre-incubated at 50 °C for 2 min, 95 °C for 10 min, followed by 40 amplification cycles of 95 °C for 15 s and 60 °C for 1 min. Comparisons of gene expression were performed using QuantStudio^TM^ Software v1.5.2 (Applied biosystems, Thermo Fisher Scientific) by the 2^−△△Ct^ method using human HPRT1 or mouse ubiquitin (UBQ) as internal control—selected as the two most stable genes per cell line in our experimental conditions. The dissociation curve for each amplification reaction (melting curve) was analyzed to confirm the absence of non-specific PCR products.

**Table 1:** List of primers.

Gene	Forward Primer	Reverse Primer
mTNFα	5′ATGA GCA CAG AAA GCA TGA3′	5′ AGT AGA CAG AAG AGC GTG GT 3′
mIRF5	5′ CCT ACA GAA CCA CTC TTG CC 3’	5’ CCT TGT GGG TTG CTG ATG GT 3’
mMYD88	5′ CAT GTT CTC CAT ACC CTT GGT 3′	5′ CAA ACT GCG AGT GGG GTC AG 3′
mTNFR1	5′ CCG GGA GAA GAG GGA TAG CTT 3′	5′ TCG GAC AGT CAC TCA CCA AGT 3′
mUBQ	5′ GAC AGG CAA GAC CAT CAC 3′	5′ TCT GAG GCG AAG GAC TAA G 3′
hTNFα	5′ CTC TTC TGC CTG CTG CAC TTT 3′	5′ ATG GGC TAC AGG CTT GTC ACT 3′
hHO−1	5′ TCC TGG CTC AGC CTC AAA TG 3′	5′ CGT TAA ACA CCT CCC TCC CC 3′
hIRF5	5′ GGG CTT CAA TGG GTC AAC G 3′	5′ GCC TTC GGT GTA TTT CCC TG 3′
hHPRT1	5′ CCT GGC GTC GTG ATT AGT GA 3′	5′ CGA GCA AGA CGT TCA GTC CT 3′

### 2.6. Bilirubin Measurement

Intracellular bilirubin generation was analyzed by transfecting RAW264.7 cells with a plasmid coding for the UnaG protein [48]. pUNAG was from by RIKEN BioResource Center, DNA Bank, Tsukuba Ibaraki, Japan.

For our experiments, cells were plated at a density of 30,000 cells/well in a 96-well plate and transfected with pUNAG plasmid by using Lipofectamine 2000 (Invitrogen—Life technologies) according to the manufacturer’s instruction. After 24 h of transfection, cells were treated as needed. At the end of treatments, the cell medium was discarded, and 50 µL/well of DMSO was added to solubilize the cells. Fluorescence was recorded in a multiwell-plate reader (CLARIOstar BMG LabTech, Germany) (ex 485-em 520) and normalized on protein cellular content.

### 2.7. Statistical Analysis

Data were analyzed by using Graph Pad Prism software (Version Prism 10, San Diego, CA, USA). The mean value ± SEM resulting from at least three experiments was calculated. One-way analysis of variance (ANOVA) and Tukey’s multiple comparison tests were applied when comparing more than three groups. A *p*-value < 0.05 was considered significant.

## 3. Results

### 3.1. HO-1 Induction Limits the Expression of TNFα in HMC3 Cells Treated with LPS

**Figure 1:**
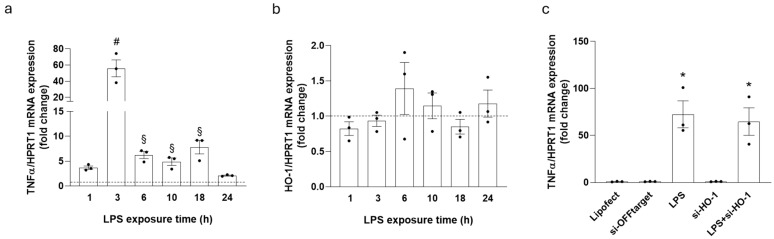
LPS-induced TNFα mRNA expression at 3 h is independent of HO-1 protein expression. (**a**,**b**) RT-qPCR time-course analysis (1–24 h) of TNFα (**a**) and HO-1 (**b**) mRNA expression in HMC3 cells exposed to 100 ng/mL LPS. The dashed line indicates baseline control levels. (**c**) RT-qPCR analysis of TNFα mRNA levels in HO-1-silenced HMC3 cells following a 3 h exposure to 100 ng/mL LPS. Statistically significant differences are indicated: #—vs. all the others time points indicated; §—vs. 24 h; *—vs. internal controls; n = 3.

To identify the time point of maximal TNFα expression in HMC3 cells exposed to 100 ng/mL LPS, a time-course analysis was carried out by RT-qPCR. The results revealed a peak in increase after 3 h (55-fold induction), followed by a progressive decline back to baseline levels by 24 h (Figure 1a). Under the same experimental conditions, RT-qPCR analysis of HO-1 mRNA expression showed no significant modifications over time (Figure 1b). HO-1 silencing was then used to evaluate the impact of HO-1 on the maximal expression of TNFα, and RT-qPCR analysis revealed no effect on the peak of TNFα expression after 3 h of LPS exposure (Figure 1c). HO-1 mRNA expression was analyzed to confirm the efficacy of the silencing technique (Supplementary Figure S4).

**Figure 2:**
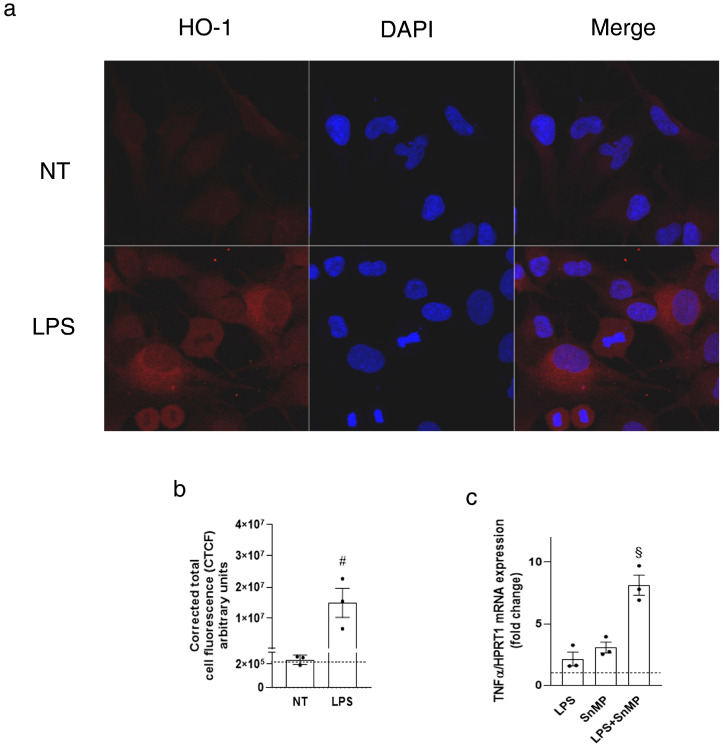
HO-1 enzymatic activity regulates LPS-induced TNFα mRNA expression at 24 h in HMC3 cells. (**a**) Confocal analysis of HO-1 protein expression in HMC3 cells exposed to 100 ng/mL LPS for 24 h. Nuclei were counterstained with DAPI. Bar = 30 µm. Images are representative of 3 independent experiments. (**b**) Quantification of fluorescence. Each value represents the average of at least 4 random fields per experimental condition. The dashed line indicates the background fluorescence intensity recorded in samples incubated with isotype-matched control antibody (rabbit normal IgG). (**c**) RT-qPCR analysis of TNFα mRNA levels in HMC3 cells exposed to 100 ng/mL LPS and/or 10 μM SnMP for 24 h. The dashed line indicates baseline control levels. Statistically significant differences are indicated: #—vs. NT; §—vs. LPS or SnMP; n = 3.

To further investigate the role of HO-1 in modulating LPS-induced TNFα expression in HMC3 cells, we examined the 24 h time point, when TNFα levels had returned to baseline. At this time-point, immunofluorescence (IF) analysis revealed a marked increase in HO-1 protein levels (Figure 2a), which was confirmed by quantification of fluorescence intensity (Figure 2b). To assess the impact of HO-1 on TNFα expression at this late stage, TNFα mRNA levels were analyzed following HO-1 inhibition. Since siRNA transient transfection failed to maintain HO-1 silencing up to 24 h of LPS treatment (Supplementary Figure S5), we utilized the pharmacological inhibitor tin mesoporphyrin IX (SnMP, 10 µM). As shown in Figure 2c, HO-1 inhibition markedly increased TNFα mRNA expression (approximately 9-fold) in LPS-treated cells compared with that in those treated with LPS alone. Notably, while HO-1 inhibition prevented TNFα expression from returning to baseline levels after 24 h of LPS treatment, it did not restore the peak expression observed at 3 h of LPS exposure (~55-fold); instead, it resulted in expression levels comparable with those recorded at 6, 10, and 18 h.

### 3.2. HO-1 Activity Limits the Expression of TNFα in RAW264.7 Cells Treated 24 h with LPS

**Figure 3:**
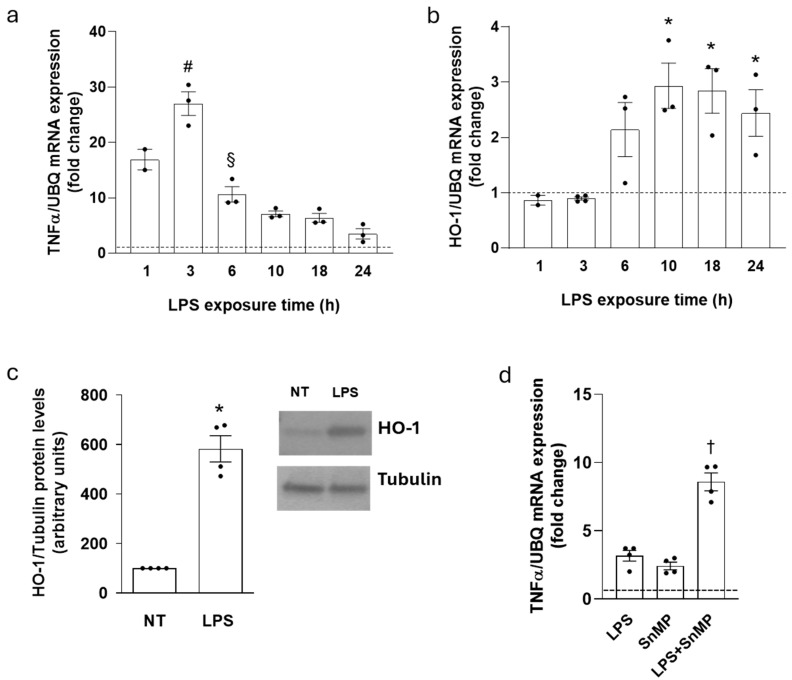
HO-1 activity regulates TNFα mRNA expression in RAW264.7 cells. (**a**,**b**) RT-qPCR time-course analysis (1–24 h) of TNFα (**a**) and HO-1 (**b**) mRNA expression in RAW264.7 cells exposed to 100 ng/mL LPS. The dashed line indicates baseline control levels. (**c**) WB analysis of HO-1 protein expression in RAW264.7 cells following a 24 h exposure to 100 ng/mL LPS. The bands show a representative blot out of 4. (**d**) RT-qPCR analysis of TNFα mRNA levels in RAW264.7 cells exposed to 100 ng/mL LPS and/or 10µM SnMP for 24 h. The dashed line indicates baseline control levels. Statistically significant differences are indicated: #—vs. all the others time points indicated; §—vs. 24 h; *—NT; †—vs. LPS or SnMP; n = 3 (**a**,**b**) or 4 (**c**,**d**). The original western blot images can be found in Supplementary Materials.

To further confirm our observations, we evaluated cell response to LPS in RAW264.7 macrophage-like cells. Similar to our findings in HMC3, time-course analysis of RAW264.7 cells exposed to LPS showed maximal TNFα mRNA induction at 3 h (about 40-fold vs. untreated cells) followed by a progressive decline to a 3-fold induction at 24 h (Figure 3a). HO-1 mRNA expression exhibited a modest, progressive induction over time (Figure 3b), also confirmed by Western blot (WB) analysis of HO-1 protein level after 24 h of LPS exposure (Figure 3c). Consistently, pharmacological inhibition of HO-1 via SnMP treatment in LPS-exposed cells significantly increased TNFα mRNA expression compared with that in cells stimulated with LPS alone (Figure 3d), even though it did not restore the maximal expression observed at 3 h LPS exposure.

Also, MYD88 and TNFR1 showed similar regulation in RAW264.7 cells (Supplementary Figure S6).

Our results highlight the endogenous role of HO-1 activity in promoting the complete resolution of LPS-induced TNFα upregulation.

### 3.3. HO-1 Limits the Expression of IRF5 in HMC3 and RAW264.7 Cells Treated with LPS

Then, we evaluated whether IRF5 could be a target for HO-1 regulation in LPS-treated cells.

**Figure 4:**
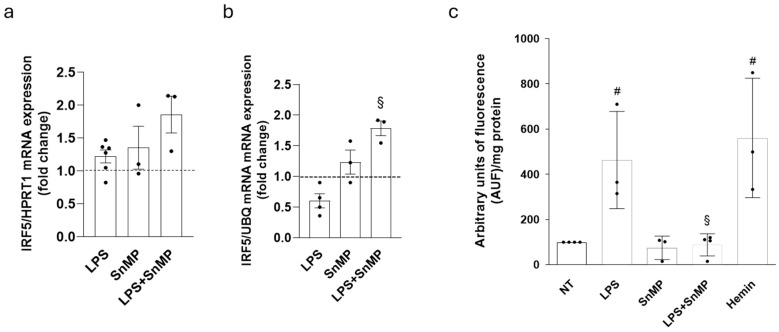
HO-1 activity regulates IRF5 mRNA expression in HMC3 and RAW264.7 cells. (**a**) RT-qPCR analysis of IRF5 mRNA in HMC3 cells exposed to 100 ng/mL LPS and/or 10 μM SnMP for 24 h. (**b**) RT-qPCR analysis of IRF5 mRNA in RAW264.7 cells treated for 24 h with 100 ng/mL LPS and/or 10 μM SnMP. (**c**) Analysis of intracellular generation of bilirubin in RAW264.7 cells exposed for 24 h to 100 ng/mL LPS and/or 10 μM SnMP. Hemin was used as a positive control. Statistically significant differences are indicated: §—vs. LPS; #—vs. NT; n = 3 (**a**,**b**) or 4 (**c**). Dashed lines represent the expression levels of control samples.

IRF5 was barely modified in terms of mRNA expression in HMC3 cells exposed to LPS for 3 or 6 h, and HO-1 silencing did not exert any modifications. However, HO-1 inhibition with SnMP increased IRF5 mRNA expression after 24 h of LPS exposure in both cell lines. This increase approached statistical significance in HMC3 cells (*p* = 0.06 vs. LPS alone, Figure 4a) and was evident in RAW264.7 cells (Figure 4b). Notably, the activity of HO-1 was measured in terms of bilirubin generation using a biomolecular approach, by overexpressing in RAW264.7 cells the fluorescent probe UnaG. As shown in Figure 4c, LPS treatment increased intracellular generation of bilirubin that was prevented by cell exposure to HO-1 inhibitor SnMP. Hemin, being a classical activator of HO-1, was used as a positive control for bilirubin generation.

### 3.4. HO-1 Inductor Hemin Reduces the Level of p-IRF5 in HMC3 Cells Treated with LPS

**Figure 5:**
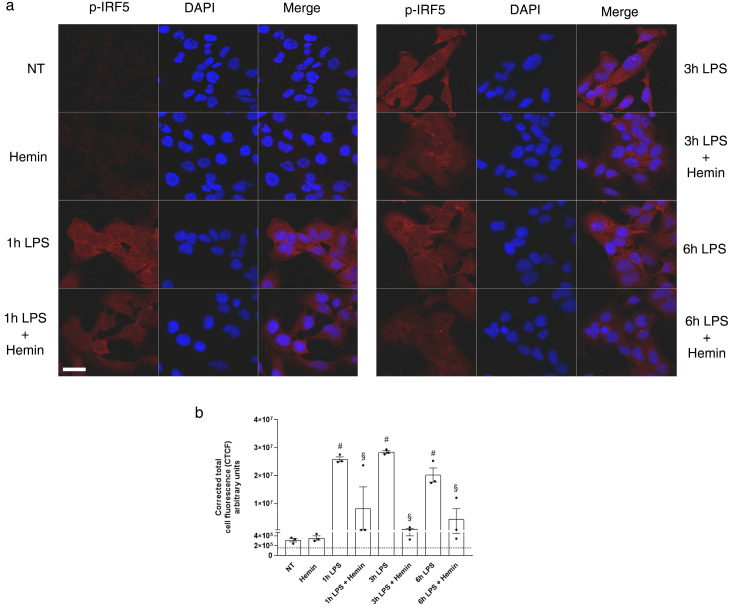
The HO-1 inducer Hemin counteracts p-IRF5 expression in HMC3 cells exposed to LPS. (**a**) Confocal immunofluorescence analysis of p-IRF5 expression in HMC3 cells treated with 5 µM Hemin and/or 100 ng/mL LPS for 1, 3, and 6 h. Nuclei were counterstained with DAPI. Scale bar = 30 µm. Images are representative of three independent experiments. (**b**) Quantification of fluorescence intensity. Each value represents the average of at least four random fields per experimental condition. The dashed line indicates the background fluorescence intensity recorded in samples incubated with non-specific normal rabbit IgG. Statistically significant differences are indicated: #—vs. NT: § vs. LPS alone; n = 3.

To confirm that IRF5 was activated upon LPS exposure, its phosphorylation state was analyzed via immunofluorescence (IF). While p-IRF5 was barely detectable in untreated cells, its levels significantly increased following 1, 3, and 6 h of LPS treatment (Figure 5). To corroborate these findings and investigate IRF5 as a potential molecular target of HO-1, cells were exposed to the HO-1 activator Hemin. As shown in Figure 5, Hemin treatment markedly reduced the LPS-induced p-IRF5 signal compared with LPS alone. Fluorescence quantification confirmed that this reduction was statistically significant.

## 4. Discussion

In this study, we provide evidence that HO-1 activity modulates LPS-induced proinflammatory activation of microglia cells acting on TNFα and IRF5. Indeed, we demonstrate that HO-1 activity endogenously promotes the complete resolution of TNFα induction after stimulation of TLR4 and is involved in the regulation of IRF5 expression. Moreover, microglia exposure to the HO-1 inducer Hemin reduces IRF5 phosphorylation, further supporting the hypothesis of a functional interplay between these pathways.

Despite increasing evidence linking the dysregulation of microglial activity to neuropathology [49,50,51,52], the molecular mechanisms underlying the loss of control of inflammatory response in CNS are far from being understood. In particular, the dysregulation of TLR-dependent signaling has been associated with neurodegenerative diseases and protein aggregation [53,54], but the molecular mechanisms are largely unknown.

In our experimental setting, we used an immortalized human microglial cell line (HMC3) that seems to overcome some limitations observed with other microglial cell lines [55] and shows some advantages compared with primary cultures from neonatal mice, which often present an immature phenotype. Consistent findings were obtained in mouse RAW264.7 cells, indicating that the results are not cell-type- or species-specific.

The two cell lines responded similarly to LPS stimulation, increasing TNFα mRNA in the short run (3 h) and progressively returning to baseline at 24 h.

Consistently with previous reports [56] we observed an increase in HO-1 protein levels in both cell lines after 24 h of LPS exposure. Interestingly, HMC3 microglial cells exhibited no alterations in HO-1 mRNA levels despite a significant upregulation of the protein, suggesting the involvement of post-transcriptional regulatory mechanisms. Conversely, in RAW264.7 macrophage-like cells, both mRNA and protein levels increased, indicating regulation at the transcriptional level. However, regardless of the specific molecular mechanisms driving the increase in HO-1 protein expression, which are not investigated in this work, our results confirm that the increased expression of HO-1 exerts a protective function. Indeed, we found that HO-1 pharmacological inhibition in both cell lines prevented TNFα transcription from coming back to the control level, maintaining a partial induction. In particular, we demonstrated that peak TNFα induction subsequent to LPS treatment was achieved at 3 h, followed by a progressive decline to control levels by 24 h. HO-1 inhibition was found not to influence peak TNFα expression but rather to modulate its complete downregulation. Our findings are in agreement with others and underline the role of HO-1 in preventing damage due to the inability both in macrophages and microglia to completely switch off the TNFα induction [56,57]. This points to the involvement of HO-1 in a mechanism whose dysregulation favors the persistence of low-grade chronic inflammation, which, while widely documented at the systemic level, could also be relevant within the framework of chronic degenerative diseases associated with neuroinflammation.

HO-1 exerts its anti-inflammatory activity through the generation of both CO [17] and bilirubin [2,7,9]. The potent anti-inflammatory activity of bilirubin [58,59,60,61] has been widely proved by studies of modest hyperbilirubinemia, which exerts strong protective activity on the cardiovascular system [19]. However, the difficulty in quantifying low-level intracellular bilirubin production remains a limiting factor in elucidating its cellular functions. Here, we used a biomolecular approach to measure HO-1-derived bilirubin [48]. Cells were transfected with a plasmid coding for a protein that becomes fluorescent when bound to bilirubin. Fluorescence quantification at the end of the experiment allows us to measure bilirubin production. Thus, we were able to verify that LPS increased intracellular generation of bilirubin similarly to Hemin and that the inhibition of HO-1 activity prevented it. However, we did not investigate its functional role in our experimental model, and the role played by CO also needs to be take into consideration to understand the molecular mechanisms underlying the anti-inflammatory effect of HO-1, and this aspect deserves future investigation.

Furthermore, we analyzed the involvement of IRF5 in our experimental model, to find a new, potentially druggable, molecular target to reduce or contain neuroinflammation.

IRF5 is a pivotal factor involved in macrophage polarization toward an M1 phenotype [62,63], and it has been found to be involved in multifactorial diseases linked to dysregulation of inflammation, including cancer progression [64,65], cardiovascular diseases [66,67] and neurological diseases [68,69].

Notably, in our experimental model, HO-1 inhibition at the latest time point of LPS treatment not only prevented TNFα from returning to baseline but also increased the mRNA expression of IRF5. Based on these findings, we cannot determine whether the effect of HO-1 on IRF5 is mediated through direct targeting or reflects a general amplification of the downstream TLR4 signaling pathway; therefore, both possibilities warrant further investigation.

Thereafter, we focused on the earliest time window to analyze whether the exogenous induction of HO-1 could modulate IRF5. We demonstrated that IRF5 is activated downstream of LPS-dependent TLR4 activation in the human microglia cell line by analyzing the expression of p-IRF5. Phosphorylation of IRF5 is, indeed, an early signal needed for IRF5 to dimerize in the cytosol and move into the nucleus, starting the transcription of target genes, and we provided evidence of IRF5 phosphorylation at 1, 3 and 6 h of LPS exposure.

Next, we demonstrated that HO-1 induction obtained by exposing cells to Hemin, can efficiently counteract p-IRF5 expression induced by LPS. Hemin is a strong inducer of HO-1 [70]; we used it at a relatively low dose, verifying the absence of toxicity due to iron exposure and the induction of HO-1. We also had confirmation in RAW264.7 cells that Hemin efficiently increased intracellular generation of bilirubin.

Notably, HO-1 and IRF5 exhibit opposing associations with aging and degenerative diseases; while HO-1 undergoes a loss of function, IRF5 becomes activated, thereby increasing susceptibility to damage and hyperinflammation [45,71,72,73]. Their role in microglial dysfunction and neuronal loss is well-recognized. While the underlying molecular mechanisms remain to be fully elucidated, our findings suggest a possible crosstalk between these molecules.

**Figure 6:**
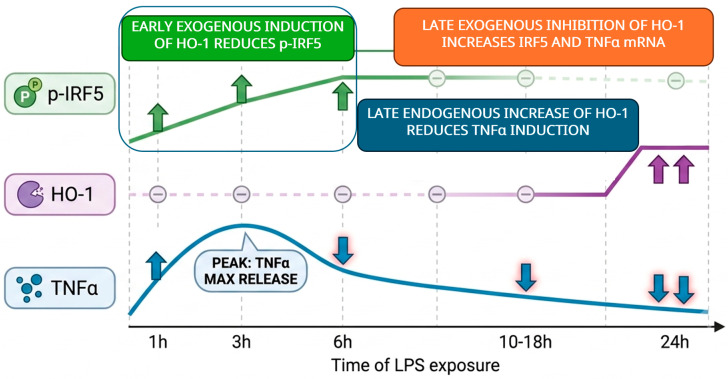
Schematic representation of the proposed mechanism and main conclusions. The diagram illustrates the time-dependent modulation of TNFα mRNA, HO-1 protein levels, and p-IRF5 expression following LPS exposure. The annotations highlight the interactions among the analyzed pathways.

In Figure 6 the main results and conclusions are presented.

## 5. Limitations of the Study

It is important to note that this work was performed on established cell lines. While this model was instrumental in uncovering a possible crosstalk among the investigated pathways and significantly facilitates an experimental approach that can often be technically challenging in more-advanced systems, such as hIPSc-derived cells, confirming these data in models that more closely recapitulate normal human physiology is essential to bolster their clinical relevance and applicability to humans.

Furthermore, the role of IRF5 in our context needs to be further validated by techniques other than immune fluorescence, for instance, by using cell fractioning to prove nuclear translocation or analyzing its DNA binding. Also, future studies should explore how HO-1 and IRF5 interact, analyzing the role of bilirubin or CO as molecular mediators of the observed effects and determining whether the interaction is direct or occurs via shared upstream regulators.

## 6. Conclusions

This work provides new insights into the role of HO-1 in endogenous regulation of TLR4-dependent responses and the possible interaction with IRF5. Our data suggests new molecular pathways to be investigated to understand and counteract microglial dysregulation.

## Data Availability

The original contributions presented in this study are included in the article/Supplementary Material. Further inquiries can be directed to the corresponding author.

## Supplementary Materials

The following supporting information can be downloaded at https://www.mdpi.com/article/10.3390/biom16071028/s1: Figure S1. MTT assays of HMC3 (a) and RAW264.7 cells (b) exposed to indicated treatments for 24 h. Dashed line represents untreated cells. No statistically significant differences observed. n = 3. Figure S2. Confocal immunofluorescence of HO-1 in HMC3 cells to prove signal specificity. (a) The images showed HMC3 cells untreated or exposed to 5 μM Hemin, 5 μM Sulforaphane (SFN) and 100 μM diethyl maleate (DEM) for 24 h analyzed following the IF protocol described in Materials and Methods. To confirm signal specificity some samples were incubated with normal rabbit IgG (1:1000, Millipore, 12-370) instead of specific antibody and revealed minimal signals. Nuclei were counterstained with DAPI. Scale bar = 30 μm. Images are representative of 3 independent experiments. (b) Quantification of fluorescence intensity. Each value represents the average of at least four random fields per experimental condition. The dashed line indicates the background fluorescence intensity recorded in samples incubated with non-specific normal rabbit IgG. Statistically significant differences are indicated: #—vs. NT; n = 3. Figure S3. Confocal immunofluorescence of p-IRF5 in HMC3 cells to prove signal specificity. (a) The images showed HMC3 cells untreated or exposed to 100 ng/mL LPS for 2 h analyzed following the IF protocol described in Materials and Methods. To confirm signal specificity some samples were incubated with normal rabbit IgG (1:1000, Millipore, 12-370) instead of specific antibody and revealed minimal signals. Nuclei were counterstained with DAPI. Scale bar = 30 μm. Images are representative of 3 independent experiments. (b) Quantification of fluorescence intensity. Each value represents the average of at least four random fields per experimental condition. The dashed line indicates the background fluorescence intensity recorded in samples incubated with non-specific normal rabbit IgG. Statistically significant differences are indicated: #—vs. NT; n = 3. Figure S4. RT-qPCR analysis of HO-1 mRNA in cells silenced for HO-1 and exposed to 100 ng/mL LPS for 3 h. Statistically significant differences are indicated: #—vs. Lipofect; n = 3. Figure S5. RT-qPCR analysis of HO-1 mRNA in cells silenced for HO-1 and exposed to 100 ng/mL LPS for 24 h. No statistically significant differences observed. n = 3. Figure S6. RT-qPCR analysis of MYD88 (a) and TNFR1 (b) mRNA in cells exposed to 100 ng/mL LPS and/or 10 μM SnMP for 24 h. Statistically significant differences are indicated: §—vs. LPS; n = 5 (a) and 4 (b). Supplementary Materials S2: The original western blot images.

## Abbreviations

Bach 1	BTB domain and CNC homolog 1
BCA	Bicinchoninic acid assay
BVR	Biliverdin reductase
CO	Carbon monoxide
CNS	Central nervous system
DNA	Deoxyribonucleic acid
HO-1	Heme oxygenase-1
IRF5	Interferon regulator factor 5
LPS	Lipopolysaccharide
MyD88	Myeloid differentiation primary response protein 88
NF-kB	Nuclear factor-kB
NRF2	Nuclear factor erythroid 2-related factor
PCR	Polymerase chain reaction
RNA	Ribonucleic acid
SnMP	Tin mesoporphyrin IX
Th1 and 2	T helper 1 and 2
TLR	Toll-like receptor
TNFα	Tumor necrosis factor alpha
TNFR1	Tumor necrosis factor receptor 1
Treg	Regulatory T cell

## References

[1] Loboda A., Damulewicz M., Pyza E., Jozkowicz A., Dulak J. (2016). Role of Nrf2/HO-1 System in Development, Oxidative Stress Response and Diseases: An Evolutionarily Conserved Mechanism. Cell. Mol. Life Sci..

[2] Paine A., Eiz-Vesper B., Blasczyk R., Immenschuh S. (2010). Signaling to Heme Oxygenase-1 and Its Anti-Inflammatory Therapeutic Potential. Biochem. Pharmacol..

[3] Ryter S.W., Choi A.M.K. (2005). Heme Oxygenase-1: Redox Regulation of a Stress Protein in Lung and Cell Culture Models. Antioxid. Redox Signal..

[4] Maines M.D. (1988). Heme Oxygenase: Function, Multiplicity, Regulatory Mechanisms, and Clinical Applications. FASEB J..

[5] Yanatori I., Richardson D.R., Toyokuni S., Kishi F. (2020). The New Role of Poly (rC)-Binding Proteins as Iron Transport Chaperones: Proteins That Could Couple with Inter-Organelle Interactions to Safely Traffic Iron. Biochim. Biophys. Acta (BBA) Gen. Subj..

[6] Duckers H.J., Boehm M., True A.L., Yet S.-F., San H., Park J.L., Clinton Webb R., Lee M.-E., Nabel G.J., Nabel E.G. (2001). Heme Oxygenase-1 Protects against Vascular Constriction and Proliferation. Nat. Med..

[7] He M., Nitti M., Piras S., Furfaro A.L., Traverso N., Pronzato M.A., Mann G.E. (2015). Heme Oxygenase-1-Derived Bilirubin Protects Endothelial Cells against High Glucose-Induced Damage. Free Radic. Biol. Med..

[8] Wang G., Hamid T., Keith R.J., Zhou G., Partridge C.R., Xiang X., Kingery J.R., Lewis R.K., Li Q., Rokosh D.G. (2010). Cardioprotective and Antiapoptotic Effects of Heme Oxygenase-1 in the Failing Heart. Circulation.

[9] Kawamura K., Ishikawa K., Wada Y., Kimura S., Matsumoto H., Kohro T., Itabe H., Kodama T., Maruyama Y. (2005). Bilirubin from Heme Oxygenase-1 Attenuates Vascular Endothelial Activation and Dysfunction. Arterioscler. Thromb. Vasc. Biol..

[10] Naito Y., Takagi T., Higashimura Y. (2014). Heme Oxygenase-1 and Anti-Inflammatory M2 Macrophages. Arch. Biochem. Biophys..

[11] Wu Y., Ge L., He S., Yang H., Zhang Y., Zhang J., Chen D., Pang Q., Huang J. (2025). Macrophage-Specific Deletion of HO-1 Aggravates Radiation-Induced Lung Injury through an PP2A-Dependent Manner. Int. Immunopharmacol..

[12] Zhang M., Nakamura K., Kageyama S., Lawal A.O., Gong K.W., Bhetraratana M., Fujii T., Sulaiman D., Hirao H., Bolisetty S. (2018). Myeloid HO-1 Modulates Macrophage Polarization and Protects against Ischemia-Reperfusion Injury. JCI Insight.

[13] Onyiah J.C., Sheikh S.Z., Maharshak N., Steinbach E.C., Russo S.M., Kobayashi T., Mackey L.C., Hansen J.J., Moeser A.J., Rawls J.F. (2013). Carbon Monoxide and Heme Oxygenase-1 Prevent Intestinal Inflammation in Mice by Promoting Bacterial Clearance. Gastroenterology.

[14] Furfaro A.L., Ottonello S., Loi G., Cossu I., Piras S., Spagnolo F., Queirolo P., Marinari U.M., Moretta L., Pronzato M.A. (2020). HO-1 Downregulation Favors BRAFV600 Melanoma Cell Death Induced by Vemurafenib/PLX4032 and Increases NK Recognition. Int. J. Cancer.

[15] Nitti M., Ortolan J., Furfaro A.L. (2023). Role of Heme Oxygenase-1 in Tumor Immune Escape. Redox Exp. Med..

[16] Brouard S., Berberat P.O., Tobiasch E., Seldon M.P., Bach F.H., Soares M.P. (2002). Heme Oxygenase-1-Derived Carbon Monoxide Requires the Activation of Transcription Factor NF-κB to Protect Endothelial Cells from Tumor Necrosis Factor-α-Mediated Apoptosis. J. Biol. Chem..

[17] Ryter S.W., Choi A.M.K. (2016). Targeting Heme Oxygenase-1 and Carbon Monoxide for Therapeutic Modulation of Inflammation. Transl. Res..

[18] Nitti M., Furfaro A.L., Mann G.E. (2020). Heme Oxygenase Dependent Bilirubin Generation in Vascular Cells: A Role in Preventing Endothelial Dysfunction in Local Tissue Microenvironment?. Front. Physiol..

[19] Bulmer A.C., Bakrania B., Du Toit E.F., Boon A.-C., Clark P.J., Powell L.W., Wagner K.-H., Headrick J.P. (2018). Bilirubin Acts as a Multipotent Guardian of Cardiovascular Integrity: More than Just a Radical Idea. Am. J. Physiol. Heart Circ. Physiol..

[20] Barisione C., Ortona S., Ivaldo C., Righetti G., Pisa F., Mena Vera J.M., Sartini M., Palombo D., Pigozzi S., Esposito D. (2025). AhR, IRF5, and HO-1 Expression in Evaluating Carotid Atherosclerotic Plaque Vulnerability: A Pilot Observational Study. J. Am. Heart Assoc..

[21] Barisione C., Garibaldi S., Furfaro A.L., Nitti M., Palmieri D., Passalacqua M., Garuti A., Verzola D., Parodi A., Ameri P. (2016). Moderate Increase of Indoxyl Sulfate Promotes Monocyte Transition into Profibrotic Macrophages. PLoS ONE.

[22] Nitti M., Ivaldo C., Traverso N., Furfaro A.L. (2021). Clinical Significance of Heme Oxygenase 1 in Tumor Progression. Antioxidants.

[23] Nitti M., Piras S., Brondolo L., Marinari U.M., Pronzato M.A., Furfaro A.L. (2018). Heme Oxygenase 1 in the Nervous System: Does It Favor Neuronal Cell Survival or Induce Neurodegeneration?. Int. J. Mol. Sci..

[24] Barone E., Di Domenico F., Mancuso C., Butterfield D.A. (2014). The Janus Face of the Heme Oxygenase/Biliverdin Reductase System in Alzheimer Disease: It’s Time for Reconciliation. Neurobiol. Dis..

[25] Cuadrado A., Rojo A.I. (2008). Heme Oxygenase-1 as a Therapeutic Target in Neurodegenerative Diseases and Brain Infections. Curr. Pharm. Des..

[26] Jazwa A., Cuadrado A. (2010). Targeting Heme Oxygenase-1 for Neuroprotection and Neuroinflammation in Neurodegenerative Diseases. Curr. Drug Targets.

[27] Mancuso C. (2022). The Brain Heme Oxygenase/Biliverdin Reductase System as a Target in Drug Research and Development. Expert Opin. Ther. Targets.

[28] Biber K., Neumann H., Inoue K., Boddeke H.W.G.M. (2007). Neuronal ‘On’ and ‘Off’ Signals Control Microglia. Trends Neurosci..

[29] Dheen S.T., Kaur C., Ling E.-A. (2007). Microglial Activation and Its Implications in the Brain Diseases. Curr. Med. Chem..

[30] Loane D.J., Kumar A. (2016). Microglia in the TBI Brain: The Good, the Bad, and the Dysregulated. Exp. Neurol..

[31] Philips T., Robberecht W. (2011). Neuroinflammation in Amyotrophic Lateral Sclerosis: Role of Glial Activation in Motor Neuron Disease. Lancet Neurol..

[32] Alfonso-Loeches S., Pascual-Lucas M., Blanco A.M., Sanchez-Vera I., Guerri C. (2010). Pivotal Role of TLR4 Receptors in Alcohol-Induced Neuroinflammation and Brain Damage. J. Neurosci..

[33] Fellner L., Irschick R., Schanda K., Reindl M., Klimaschewski L., Poewe W., Wenning G.K., Stefanova N. (2013). Toll-like Receptor 4 Is Required for α-Synuclein Dependent Activation of Microglia and Astroglia. GLIA.

[34] Jack C.S., Arbour N., Manusow J., Montgrain V., Blain M., McCrea E., Shapiro A., Antel J.P. (2005). TLR Signaling Tailors Innate Immune Responses in Human Microglia and Astrocytes. J. Immunol..

[35] Tanga F.Y., Nutile-McMenemy N., DeLeo J.A. (2005). The CNS Role of Toll-like Receptor 4 in Innate Neuroimmunity and Painful Neuropathy. Proc. Natl. Acad. Sci. USA.

[36] Kim S., Becker J., Bechheim M., Kaiser V., Noursadeghi M., Fricker N., Beier E., Klaschik S., Boor P., Hess T. (2014). Characterizing the Genetic Basis of Innate Immune Response in TLR4-Activated Human Monocytes. Nat. Commun..

[37] O’Neill L.A.J., Bowie A.G. (2007). The Family of Five: TIR-Domain-Containing Adaptors in Toll-like Receptor Signalling. Nat. Rev. Immunol..

[38] Tang L., Chen B., Ma B., Nie S. (2014). Association between IRF5 Polymorphisms and Autoimmune Diseases: A Meta-Analysis. Genet. Mol. Res..

[39] Lee Y.H., Bae S.-C., Choi S.J., Ji J.D., Song G.G. (2012). Genome-Wide Pathway Analysis of Genome-Wide Association Studies on Systemic Lupus Erythematosus and Rheumatoid Arthritis. Mol. Biol. Rep..

[40] Yang Y., Zhang C., Jing D., He H., Li X., Wang Y., Qin Y., Xiao X., Xiong H., Zhou G. (2021). IRF5 Acts as a Potential Therapeutic Marker in Inflammatory Bowel Diseases. Inflamm. Bowel Dis..

[41] Lees C.W., Barrett J.C., Parkes M., Satsangi J. (2011). New IBD Genetics: Common Pathways with Other Diseases. Gut.

[42] He Z., Luo Y., Duan Z., Su B., Zeng W., Guo Y., Li Y., He X., Shi H., Zhou Z. (2025). IRF5 siRNA Nanoimmunotherapy: Restoring Macrophage Efferocytosis in Atherosclerosis. Circulation.

[43] Yu X., Rehman A.U., Dang L., Zhang X., Liu J., Xiong X., Chen G., Jian Z. (2025). Interferon Regulatory Factor 5: A Potential Target for Therapeutic Intervention in Inflammatory Diseases. Front. Immunol..

[44] Montilla A., Zabala A., Calvo I., Bosch-Juan M., Tomé-Velasco I., Mata P., Koster M., Sierra A., Kooistra S.M., Soria F.N. (2025). Microglia Regulate Myelin Clearance and Cholesterol Metabolism after Demyelination via Interferon Regulatory Factor 5. Cell. Mol. Life Sci..

[45] Ngwa C., Al Mamun A., Qi S., Sharmeen R., Conesa M.P.B., Ganesh B.P., Manwani B., Liu F. (2024). Central IRF4/5 Signaling Are Critical for Microglial Activation and Impact on Stroke Outcomes. Transl. Stroke Res..

[46] Piras S., Furfaro A.L., Caggiano R., Brondolo L., Garibaldi S., Ivaldo C., Marinari U.M., Pronzato M.A., Faraonio R., Nitti M. (2018). microRNA-494 Favors HO-1 Expression in Neuroblastoma Cells Exposed to Oxidative Stress in a Bach1-Independent Way. Front. Oncol..

[47] Piras S., Furfaro A.L., Piccini A., Passalacqua M., Borghi R., Carminati E., Parodi A., Colombo L., Salmona M., Pronzato M.A. (2014). Monomeric Aβ1-42 and RAGE: Key Players in Neuronal Differentiation. Neurobiol. Aging.

[48] Kumagai A., Ando R., Miyatake H., Greimel P., Kobayashi T., Hirabayashi Y., Shimogori T., Miyawaki A. (2013). XA Bilirubin-Inducible Fluorescent Protein from Eel Muscle. Cell.

[49] Kaur D., Sharma V., Deshmukh R. (2019). Activation of Microglia and Astrocytes: A Roadway to Neuroinflammation and Alzheimer’s Disease. Inflammopharmacology.

[50] Yin F., Sancheti H., Patil I., Cadenas E. (2016). Energy Metabolism and Inflammation in Brain Aging and Alzheimer’s Disease. Free Radic. Biol. Med..

[51] Réus G.Z., Fries G.R., Stertz L., Badawy M., Passos I.C., Barichello T., Kapczinski F., Quevedo J. (2015). The Role of Inflammation and Microglial Activation in the Pathophysiology of Psychiatric Disorders. Neuroscience.

[52] Castro-Gomez S., Heneka M.T. (2024). Innate Immune Activation in Neurodegenerative Diseases. Immunity.

[53] Leitner G.R., Wenzel T.J., Marshall N., Gates E.J., Klegeris A. (2019). Targeting Toll-like Receptor 4 to Modulate Neuroinflammation in Central Nervous System Disorders. Expert Opin. Ther. Targets.

[54] Heidari A., Yazdanpanah N., Rezaei N. (2022). The Role of Toll-like Receptors and Neuroinflammation in Parkinson’s Disease. J. Neuroinflamm..

[55] Dello Russo C., Cappoli N., Coletta I., Mezzogori D., Paciello F., Pozzoli G., Navarra P., Battaglia A. (2018). The Human Microglial HMC3 Cell Line: Where Do We Stand? A Systematic Literature Review. J. Neuroinflamm..

[56] Akamatsu Y., Pagan V.A., Hanafy K.A. (2020). The Role of TLR4 and HO-1 in Neuroinflammation after Subarachnoid Hemorrhage. J. Neurosci. Res..

[57] Song Y., Shi Y., Ao L.-H., Harken A.H., Meng X.-Z. (2003). TLR4 Mediates LPS-Induced HO-1 Expression in Mouse Liver: Role of TNF-α and IL-1β. World J. Gastroenterol..

[58] Keshavan P., Deem T.L., Schwemberger S.J., Babcock G.F., Cook-Mills J.M., Zucker S.D. (2005). Unconjugated Bilirubin Inhibits VCAM-1-Mediated Transendothelial Leukocyte Migration. J. Immunol..

[59] Mazzone G.L., Rigato I., Ostrow J.D., Bossi F., Bortoluzzi A., Sukowati C.H.C., Tedesco F., Tiribelli C. (2009). Bilirubin Inhibits the TNFα-Related Induction of Three Endothelial Adhesion Molecules. Biochem. Biophys. Res. Commun..

[60] Liu Y., Li P., Lu J., Xiong W., Oger J., Tetzlaff W., Cynader M. (2008). Bilirubin Possesses Powerful Immunomodulatory Activity and Suppresses Experimental Autoimmune Encephalomyelitis. J. Immunol..

[61] Rocuts F., Zhang X., Yan J., Yue Y., Thomas M., Bach F.H., Czismadia E., Wang H. (2010). Bilirubin Promotes de Novo Generation of T Regulatory Cells. Cell Transplant..

[62] Krausgruber T., Blazek K., Smallie T., Alzabin S., Lockstone H., Sahgal N., Hussell T., Feldmann M., Udalova I.A. (2011). IRF5 Promotes Inflammatory Macrophage Polarization and T H1-TH17 Responses. Nat. Immunol..

[63] Khoyratty T.E., Udalova I.A. (2018). Diverse Mechanisms of IRF5 Action in Inflammatory Responses. Int. J. Biochem. Cell Biol..

[64] Roberts B.K., Li D.I., Somerville C., Matta B., Jha V., Steinke A., Brune Z., Blanc L., Soffer S.Z., Barnes B.J. (2024). IRF5 Suppresses Metastasis through the Regulation of Tumor-Derived Extracellular Vesicles and Pre-Metastatic Niche Formation. Sci. Rep..

[65] Roberts B.K., Collado G., Barnes B.J. (2024). Role of Interferon Regulatory Factor 5 (IRF5) in Tumor Progression: Prognostic and Therapeutic Potential. Biochim. Biophys. Acta Rev. Cancer.

[66] Edsfeldt A., Swart M., Singh P., Dib L., Sun J., Cole J.E., Park I., Al-Sharify D., Persson A., Nitulescu M. (2022). Interferon Regulatory Factor-5-Dependent CD11c+macrophages Contribute to the Formation of Rupture-Prone Atherosclerotic Plaques. Eur. Heart J..

[67] Seneviratne A.N., Edsfeldt A., Cole J.E., Kassiteridi C., Swart M., Park I., Green P., Khoyratty T., Saliba D., Goddard M.E. (2017). Interferon Regulatory Factor 5 Controls Necrotic Core Formation in Atherosclerotic Lesions by Impairing Efferocytosis. Circulation.

[68] Al Mamun A., Chauhan A., Qi S., Ngwa C., Xu Y., Sharmeen R., Hazen A.L., Li J., Aronowski J.A., McCullough L.D. (2020). Microglial IRF5-IRF4 Regulatory Axis Regulates Neuroinflammation after Cerebral Ischemia and Impacts Stroke Outcomes. Proc. Natl. Acad. Sci. USA.

[69] Fan Z., Zhao S., Zhu Y., Li Z., Liu Z., Yan Y., Tian J., Chen Y., Zhang B. (2020). Interferon Regulatory Factor 5 Mediates Lipopolysaccharide-Induced Neuroinflammation. Front. Immunol..

[70] Foresti R., Bains S.K., Pitchumony T.S., de Castro Brás L.E., Drago F., Dubois-Randé J.-L., Bucolo C., Motterlini R. (2013). Small Molecule Activators of the Nrf2-HO-1 Antioxidant Axis Modulate Heme Metabolism and Inflammation in BV2 Microglia Cells. Pharmacol. Res..

[71] Patriarca S., Furfaro A.L., Cosso L., Pesce Maineri E., Balbis E., Domenicotti C., Nitti M., Cottalasso D., Marinari U.M., Pronzato M.A. (2007). Heme Oxygenase 1 Expression in Rat Liver during Ageing and Ethanol Intoxication. Biogerontology.

[72] Ngwa C., Al Mamun A., Qi S., Sharmeen R., Xu Y., Liu F. (2022). Regulation of Microglial Activation in Stroke in Aged Mice: A Translational Study. Aging.

[73] Sethi P., Mehan S., Khan Z., Maurya P.K., Kumar N., Kumar A., Tiwari A., Sharma T., Das Gupta G., Narula A.S. (2025). The SIRT-1/Nrf2/HO-1 Axis: Guardians of Neuronal Health in Neurological Disorders. Behav. Brain Res..

