# Supplementary material for: Heme Oxygenase-1 Contributes to Dampening Proinflammatory Activation in the Human Microglial Cell Line HMC3 and Controls the Transcription Factor IRF5
Source: Biomolecules. 2026 Jul 14;16(7):1028. doi: 10.3390/biom16071028 (PMC13406587; doi:10.3390/biom16071028)
Supplement: Supplementary file 1 [file biomolecules-16-01028-s001.zip › biomolecules-4209274-supplementary.pdf]

Article

Heme Oxygenase-1 Contributes to Dampening Proinflammatory Activation in Human Microglial Cells HMC3 and Controls the Transcription Factor IRF5

Furfaro et al.

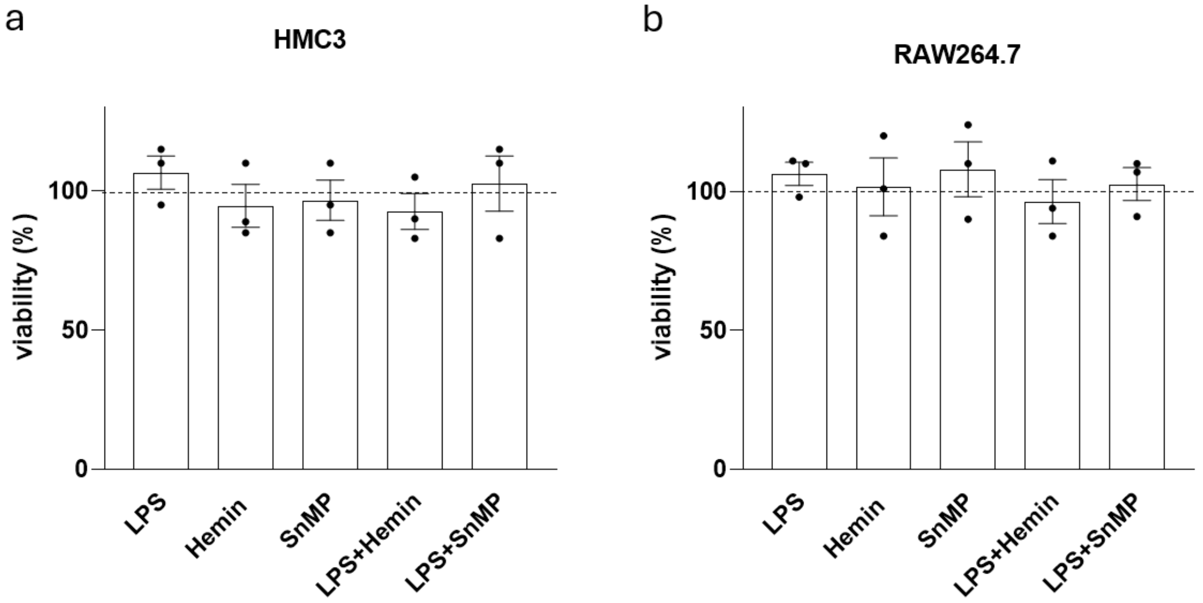

Supplementary Figure S1. MTT assays of HMC3 (a) and RAW264.7 cells (b) exposed to indicated treatments for 24h. Dashed line represents untreated cells. No statistically significant differences observed. n=3.

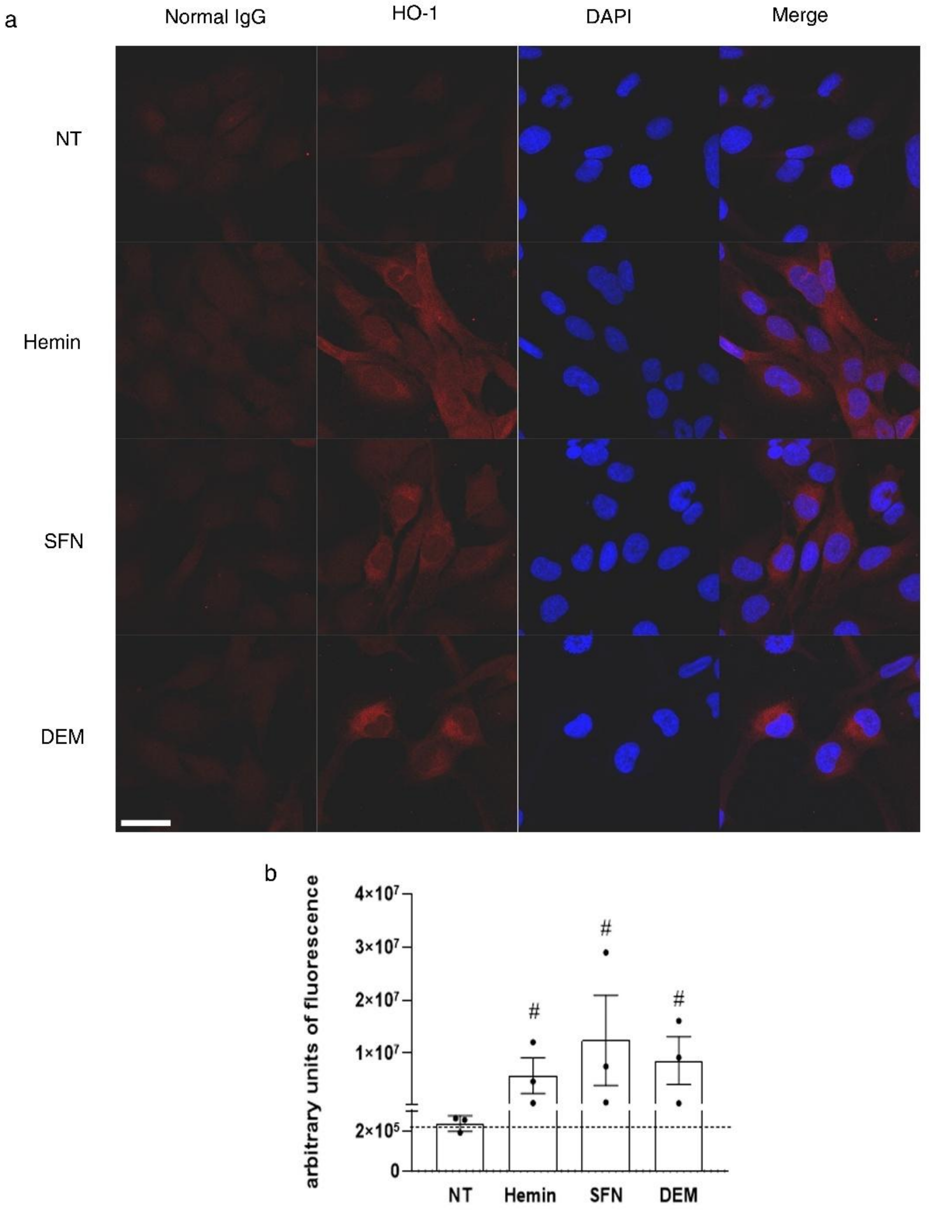

Supplementary Figure S2. Confocal immunofluorescence of HO-1 in HMC3 cells to prove signal specificity. (a) The images showed HMC3 cells untreated or exposed to 5μM Hemin, 5μM Sulforaphane (SFN) and 100μM diethyl maleate (DEM) for 24h analyzed following the IF protocol described in Materials and Methods. To confirm signal specificity some samples were incubated with normal rabbit IgG (1:1000, Millipore, 12-370) instead of specific antibody and revealed minimal signals. Nuclei were counterstained with DAPI. Scale bar=30μm. Images are representative of 3 independent experiments. (b) Quantification of fluorescence intensity. Each value represents the average of at least four random fields per experimental condition. The dashed line indicates the background fluorescence intensity recorded in samples incubated with non-specific normal rabbit IgG. Statistically significant differences are indicated: # -vs NT; n=3.

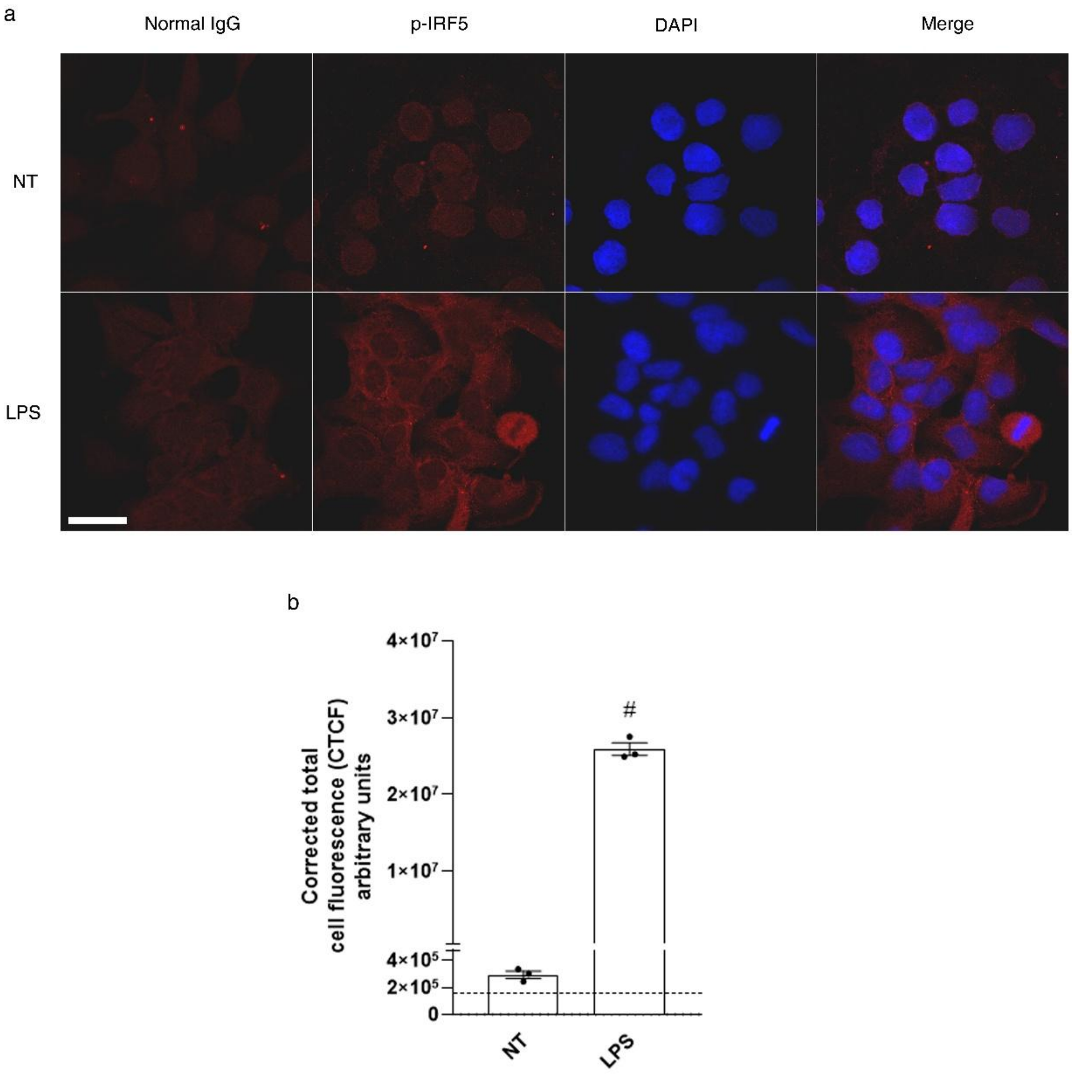

Supplementary Figure S3. Confocal immunofluorescence of p-IRF5 in HMC3 cells to prove signal specificity. (a) The images showed HMC3 cells untreated or exposed to 100ng/ml LPS for 2h analyzed following the IF protocol described in Materials and Methods. To confirm signal specificity some samples were incubated with normal rabbit IgG (1:1000, Millipore, 12-370) instead of specific antibody and revealed minimal signals. Nuclei were counterstained with DAPI. Scale bar=30μm. Images are representative of 3 independent experiments.
(b) Quantification of fluorescence intensity. Each value represents the average of at least four random fields per experimental condition. The dashed line indicates the background fluorescence intensity recorded in samples incubated with non-specific normal rabbit IgG. Statistically significant differences are indicated: # - vs NT; n=3.

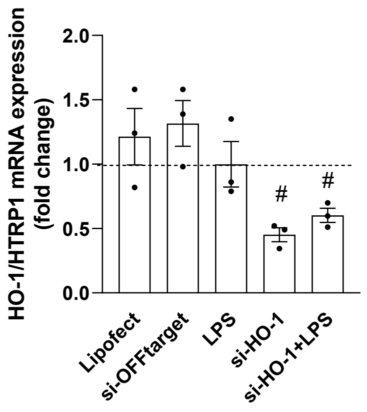

Supplementary Figure S4. RT-qPCR analysis of HO-1 mRNA in cells silenced for HO-1 and exposed to 100ng/ml LPS for 3h. Statistically significant differences are indicated: # - vs Lipofect; n=3.

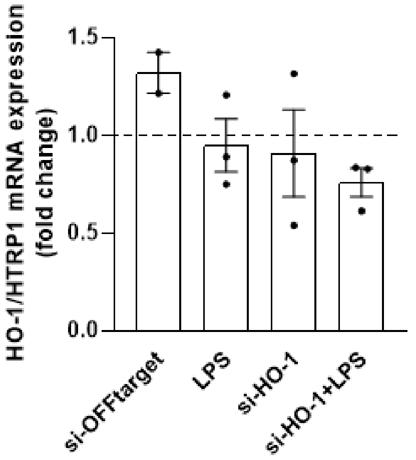

Supplementary Figure S5. RT-qPCR analysis of HO-1 mRNA in cells silenced for HO-1 and exposed to 100ng/ml LPS for 24h. No statistically significant differences observed. n=3.

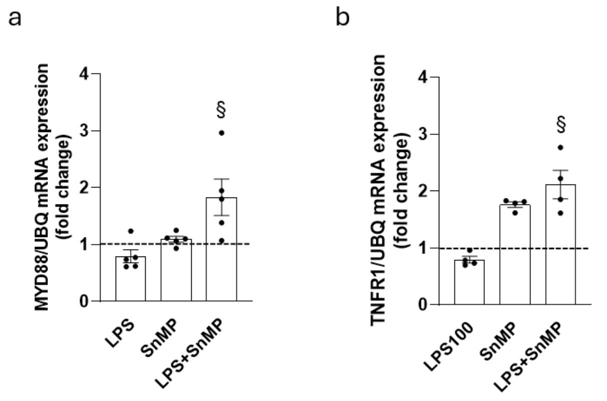

Supplementary Figure S6. RT-qPCR analysis of MYD88 (a) and TNFR1 (b) mRNA in cells exposed to 100ng/ml LPS and/or 10 μM SnMP for 24h. Statistically significant differences are indicated: § - vs LPS; n=5 (a) and 4 (b).
