# Supplementary figures and images for: Heme Oxygenase-1 Contributes to Dampening Proinflammatory Activation in the Human Microglial Cell Line HMC3 and Controls the Transcription Factor IRF5
Source: Biomolecules. 2026 Jul 14;16(7):1028. doi: 10.3390/biom16071028 (PMC13406587; doi:10.3390/biom16071028)

Original Blots used for Figure 3b

NT LPS

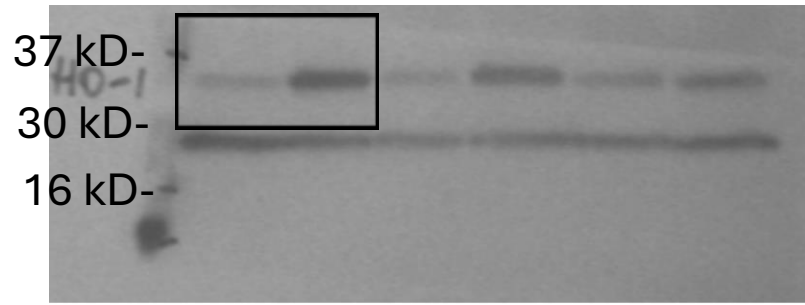

NT LPS

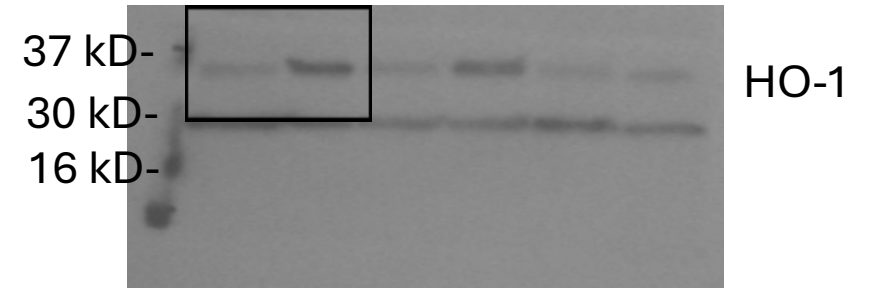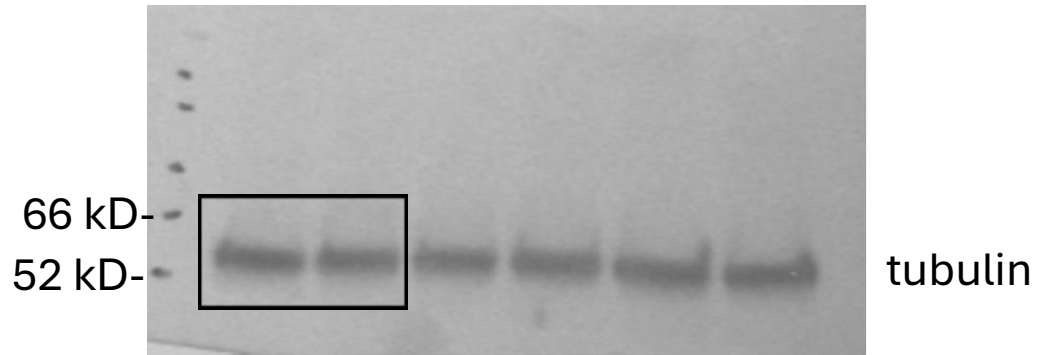

NT LPS

#1

This has been shown in figure3b

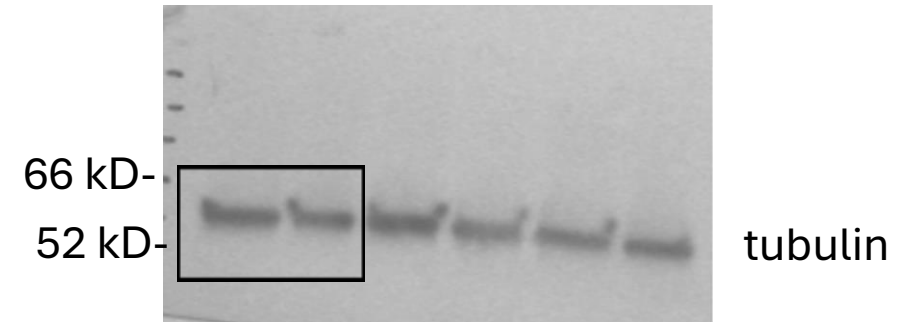

NT LPS

#2

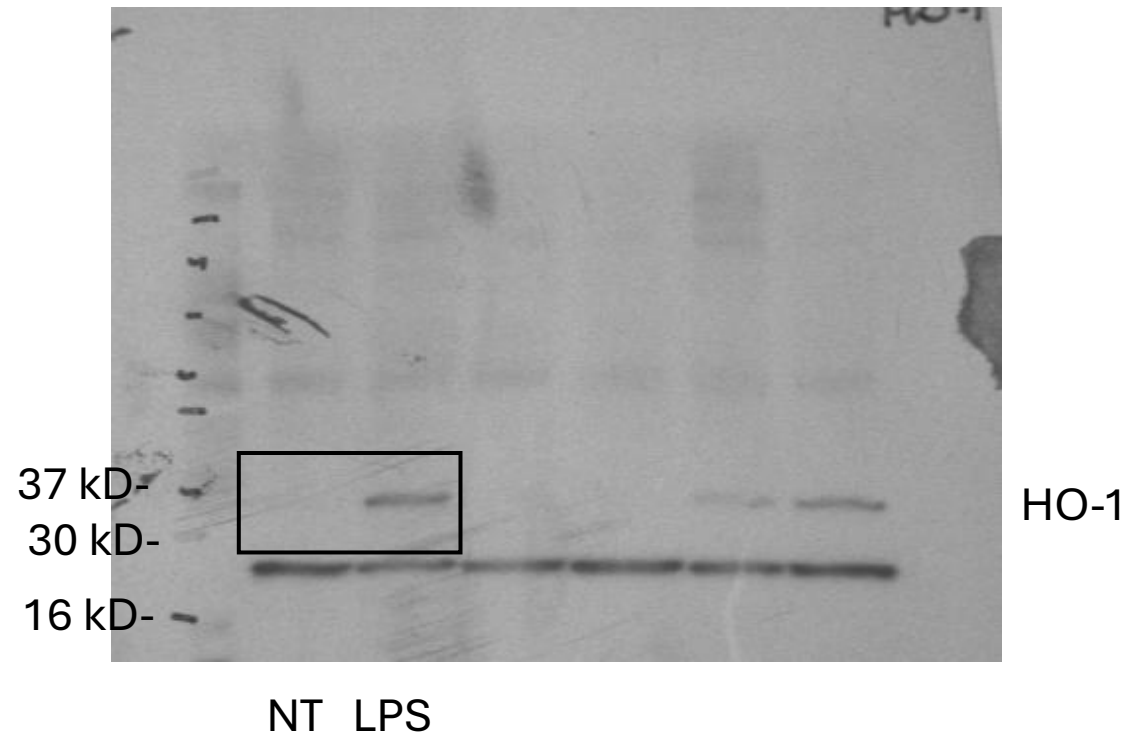

#3

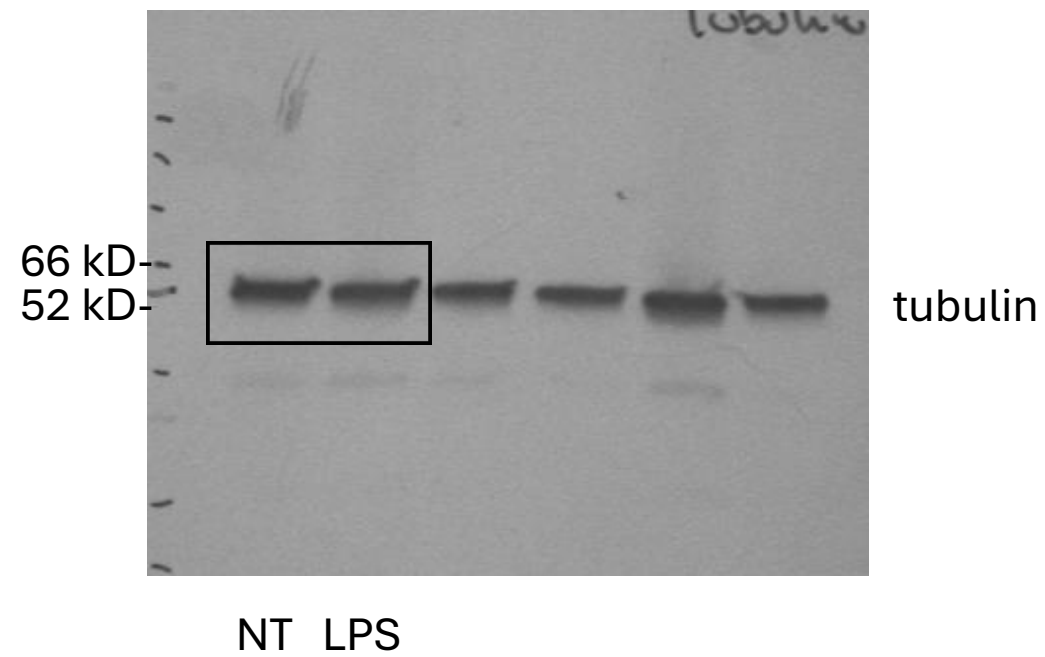

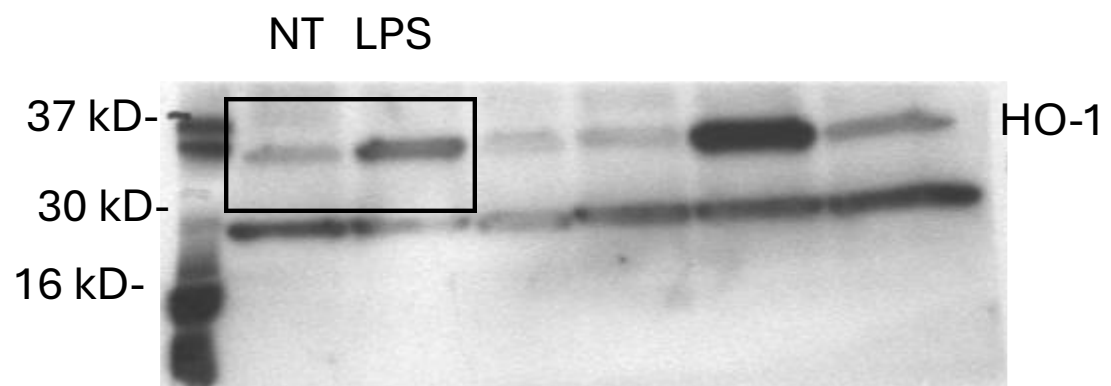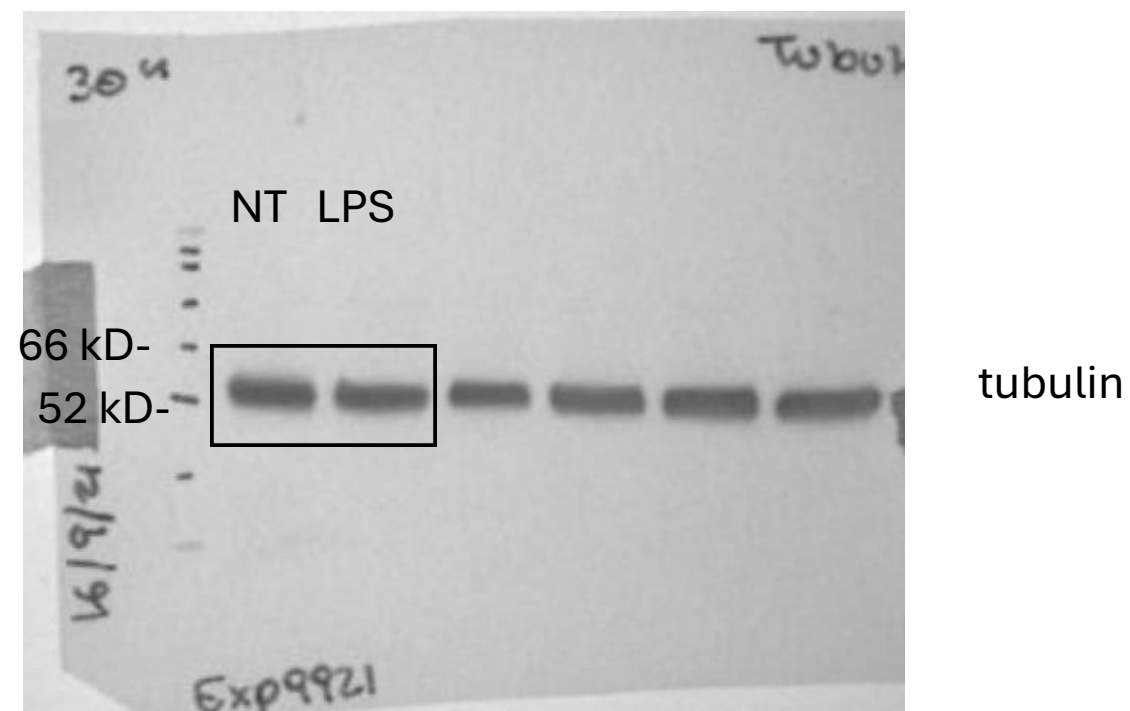

#4

Supplement: Supplementary file 1 [file biomolecules-16-01028-s001.zip › biomolecules-4209274 original fig.pdf]
